# THE VALUE OF BALANCE CONFIDENCE IN OLDER ADULT FALL RISK ASSESSMENT

**DOI:** 10.1093/geroni/igac059.147

**Published:** 2022-12-20

**Authors:** Tiffany Hughes, Edmund Ickert, Garrett Kellar, Lucy Kerns, Cara Berg-Carramusa

**Affiliations:** Youngstown State University, Beaver, Pennsylvania, United States; Youngstown State University, Youngstown, Ohio, United States; Youngstown State University, Youngstown, Ohio, United States; Youngstown State University, Youngstown, Ohio, United States; Youngstown State University, Youngstown, Ohio, United States

## Abstract

Fall risk assessment traditionally focuses on objective physical performance. Balance confidence, a subjective measure of physical function, may provide important information to better predict fall risk and guide assessment and intervention strategies. This study examines the associations of balance confidence congruency with physical performance measures and fall occurrence. One-hundred-fifty-five community-dwelling adults aged 60 and over completed a comprehensive fall risk assessment including physical performance tests (timed up and go, 4-stage balance, 30-second chair stand, and 4-M gait speed), activities-specific balance confidence (ABC) scale, and self-reported falls in the past year. Four groups were created based on congruency between balance confidence (low vs. high) and each physical performance measure or overall fall risk category (at fall risk vs. not at fall risk, based on the STEADI tool kit). Poison regression analyses, adjusted for age and gender, tested the association between group membership and number of falls in the past year. Participants with high balance confidence and at fall risk based on 4-stage balance performance (Estimate=0.88, p < 0.001), or high balance confidence and at fall risk following the STEADI screening algorithm (Estimate=0.69, p = 0.003) were at increased risk of more falls compared to participants in the group with high balance confidence and not at fall risk. These results suggest that older adults who overestimate their balance relative to their physical performance may be at increased fall risk, and that participant subjective reporting of physical performance should be paired with objective physical performance measures to better identify older adults at fall risk.

